# A Tunisian version of the confusion assessment method for the intensive care unit (CAM-ICU): translation and validation

**DOI:** 10.1186/s12888-020-02622-z

**Published:** 2020-05-06

**Authors:** Imen Ben Saida, Saiid Kortli, Badii Amamou, Nawres Kacem, Mariem Ghardallou, Eugene Wesley Ely, Helmi Ben Saad, Mohamed Boussarsar

**Affiliations:** 1 Medical Intensive Care Unit, Farhat Hached University Hospital, 4000 Sousse, Tunisia; 2 Research Laboratory N° LR12SP09, Heart Failure, Faculty of Medicine of Sousse, University of Sousse, 4000 Sousse, Tunisia; 3 Department of Psychiatry, Fattouma Bourguiba University Hospital, 5000 Monastir, Tunisia; 4 Department of Community and Preventive Medicine, Faculty of Medicine, 4000 Sousse, Tunisia; 5 Critical Illness, Brain Dysfunction, and Survivorship (CIBS) Center, Vanderbilt University Medical Center, and the Veteran’s Affairs Tennessee Valley Geriatric Research Education and Clinical Center (GRECC), Nashville, USA; 6 University of Sousse, Faculty of medicine of Sousse, Laboratory of Physiology, Sousse, Tunisia

**Keywords:** Delirium, Critical illness, Translations, North Africa

## Abstract

**Background:**

Delirium is common in critically ill patients and it is associated with poor outcomes. In Tunisia, however, it is still underdiagnosed as there is no validated screening tool. The aim of this study was to translate and to validate a Tunisian version of the CAM-ICU.

**Methods:**

For the validation and inter-rater reliability assessment of the Tunisian CAM-ICU, two trained intensivists independently evaluated delirium in the patients admitted to the ICU between October 2017 and June 2018. All the patients consecutively admitted to the ICU for more than 24 h and having a Richmond Agitation-Sedation Scale greater than or equal to “-3” were assessed for delirium excluding those with stroke, dementia, psychosis or persistent coma. The results were compared with the reference evaluation carried out by a psychiatrist using the fifth edition of the Diagnostic and Statistical Manual of Mental Disorders (DSM-V) criteria. The inter-rater reliability was calculated using the kappa (κ) statistic. The CAM-ICU concurrent validity was assessed using Cronbach’s α coefficient, sensitivity, specificity as well as positive and negative predictive values (PPV and NPV, respectively) for the two Tunisian CAM-ICU raters.

**Results:**

The study involved 137 patients [median (IQR) age: 60 [49–68] years, male sex (*n* = 102), invasive mechanical ventilation (*n* = 49)]. Using the DSM-V criteria evaluations, 46 patients were diagnosed with delirium. When applying the Tunisian version of the CAM-ICU, 38(27.7%) patients were diagnosed with delirium for the first rater and 45(32.6%) patients for the second one. The Tunisian CAM-ICU showed a very-high inter-rater reliability for both intensivists (κ = 0.844, *p* < 0.001). Using the DSM-V rater as the reference standard, the sensitivity of the two intensivists’ evaluations was 80.4 vs. 95.7%. Specificity was 98.9% for both. The Cronbach’s α of the first and second raters’ evaluations using the Tunisian version of the CAM-ICU were 0.886 and 0.887, respectively.

**Conclusions:**

The Tunisian version of the CAM-ICU showed almost perfect validity and reliability in detecting delirium in critically ill patients. It could therefore be used in Tunisian ICUs or where Tunisian translators are available following appropriate training.

**Trial registration:**

Not applicable.

## Background

Delirium is an acute brain dysfunction characterized by fluctuating levels of disturbance in consciousness and cognition, impaired short term memory, disturbed attention and disorientation [1]. This serious problem can be detrimental to patients’ safety [2, 3]. When delirium occurs in patients admitted to an intensive care unit (ICU), it can be associated with adverse outcomes such as self-extubation, catheter removal, difficulties of weaning, prolonged stay, higher mortality rates, and consequently higher healthcare costs [4]. Given the high prevalence of delirium in ICU patients (from 20 to 80%) and given its morbidity and mortality, the Society of Critical Care Medicine (SCCM) recommends its regular assessment [5]. However, delirium remains either underdiagnosed, lately detected or even not detected [6–8]. About 24 different delirium scales are available for non-ICU population [9]. However, their use for non-verbal mechanically ventilated patients is difficult [9, 10]. According to the SCCM, the Confusion Assessment Method (CAM-ICU) and the Intensive Care Delirium Screening Checklist are the most valid and reliable tools for screening delirium in ICU patients [5, 11, 12]. The CAM-ICU is the most widely used tool for delirium assessment in ICUs [1]. It is based on the CAM designed for healthcare providers without a formal psychiatric training [13].

To the best of the authors’ knowledge, there is no validated version of the CAM-ICU in North Africa or the Maghreb region. In these areas, delirium screening in ICUs is not routinely performed by the medical staff in ICUs. In fact, most diagnoses rely only on clinical symptoms. The CAM-ICU, translated to over 26 languages, has recently been translated and validated in the Middle Eastern Arabic countries [14, 15]. However, since the Maghrebi/Tunisian dialect is completely different from modern standard Arabic or the Egyptian dialect, the extent of the aforementioned versions [14, 15] is not mutually intelligible, mostly for critically ill patients. For this reason, translation of the CAM-ICU into the Tunisian dialect seems to be a crucial step to improve delirium detection in Tunisian ICUs.

The aim of the present study was to translate the English version of the CAM-ICU into the Tunisian dialect and to assess its cultural validity and reliability on a sample of Tunisian ICU patients.

## Methods

### Ethical considerations

An agreement was obtained from Professor Ely (Vanderbilt University Medical Center, Nashville, USA), the designer of the CAM-ICU scale. The patients involved were informed about the voluntary and anonymous nature of the study. A written consent was obtained directly from each one. However, for the patients who were temporarily unable to decide for themselves, written consents were obtained from their relatives. Later, these patients were informed about the study and written consent was obtained.

### Translation and cultural adaptation procedures

The translation and cultural adaptation procedures were performed in four steps according to the protocol of the “Linguistic Validation Manual for Health Outcome Assessments” developed by MAPI institute (http://www.mapigroup.com). First, the translation was carried out from English into the Tunisian dialect by two bilingual translators (*IB* and *SK* in the authors’ list). A reconciliation of the two forward translations was performed. Secondly, a back-translation was conducted by a bilingual translator who had no information about the original version. Thirdly, a meeting involving all the development team was held to check the conformity of the back-translated text with the original one. All the differences between the original and the back-translated versions were discussed. Suggestions for the items that could be ambiguous or misunderstood were encouraged. Responses and comments were taken into consideration in the reconciled and agreed upon forward-translated version of the Tunisian CAM-ICU. Finally, the back-translated version was sent to Professor Ely for approval.

The material related to the Tunisian translated version of the CAM-ICU is currently available at the following website: www.icudelirium.org (last access: March 22, 2020).

### Delirium assessment by CAM-ICU

The CAM-ICU scale comprises four features. *Feature-1* an acute change or fluctuation in the course of the mental status. *Feature-2* inattention and it is assessed using attention screening examination letters (auditory vigilance random letter task) and pictures (visual picture recognition). *Feature-3* an altered level of consciousness evaluated using the Richmond Agitation-Sedation Scale (RASS). *Feature-4* disorganized thinking using “yes/no” questions and commands. Delirium is considered positive when features 1 and 2 plus either feature 3 or 4 are present [16].

### Pre-testing

Pre-testing was performed on a small sample of critically ill monolingual (target language, Tunisian dialect) patients. This sample was excluded from the final statistical validation group. Both CAM-ICU trained raters (*SK* and *NK* in the authors’ list) reported no difficulty or ambiguity.

### Study design

A prospective cohort study was conducted in a 9-bed medical ICU at FARHAT HACHED university hospital (Sousse, Tunisia) from October 2017 to June 2018. The average number of ICU admissions is 260 per year and the main reason for admission is acute exacerbation of chronic obstructive pulmonary disease.

### Sample size

To obtain representative and reliable data, the required sample size was estimated using the following eq. [17]: *n* = (Z_α/2_^2^ p q)/∆^2^. “***Z***_***α/2***_*”* (=1.96) was the normal deviate for a one-tailed hypothesis at a 5% level of significance; “**p**” (=0.19) was the frequency of delirium among Tunisian ICU patients in a previous study [18]; “**q**” (=0.81) was equal to “1-p”, “**∆**” (=7%) was the arbitrarily chosen precision. Using the aforementioned equation, the estimated sample size was 120 patients.

### Populations and procedure

All the patients consecutively admitted to the ICU for more than 24 h and having a RASS [19] greater than or equal to “-3” were assessed for delirium. The patients with stroke, dementia, psychosis or persistent coma were not included in this study.

The following patients’ demographic and clinical characteristics were collected: age (years), sex, addictive behaviors (smoking, alcohol abuse), underlying diseases, Charlson index [20], reasons for admission, and Simplified Acute Physiological Score (SAPS-II) [21].

Delirium detection was performed by two raters who received training on using the Tunisian version of the CAM-ICU. Rater 1 (*SK* in the authors’ list) is a well-trained resident in critical care having more than 2 years of experience. Rater 2 (*NK* in the authors’ list) is a well-trained medical intern in ICU at the time of the study. For a reference standard evaluation, a psychiatrist (*BA* in the authors’ list) applied the Diagnostic and Statistical Manual of Mental Disorders, Fifth Edition (DSM-V) criteria [22] for the delirium diagnosis. Examinations were performed less than 4 h apart. The raters were blinded to each other’s findings. Each patient was assessed once.

### Statistical analysis

The CAM-ICU inter-rater reliability was tested by comparing the Tunisian CAM-ICU rating by two raters using the Cohen’s kappa (κ) coefficient with 95% confidence interval (95% CI). “κ” coefficient was used to calculate the concordance between the two raters, defining “κ” > 0.61 as “substantial” and “κ” > 0.81 as “almost perfect” [23]. The CAM-ICU concurrent validity was assessed by calculating the internal consistency (Cronbach’s α coefficient), sensitivity, specificity as well as positive and negative predictive values (PPV and NPV, respectively) for the two Tunisian CAM-ICU raters. The calculations were based on considering the DSM-V criteria [22] as the reference standard. Data were analyzed using Epi info. Statistical significance was considered at *p* < 0.05.

## Results

**Fig. 1:**
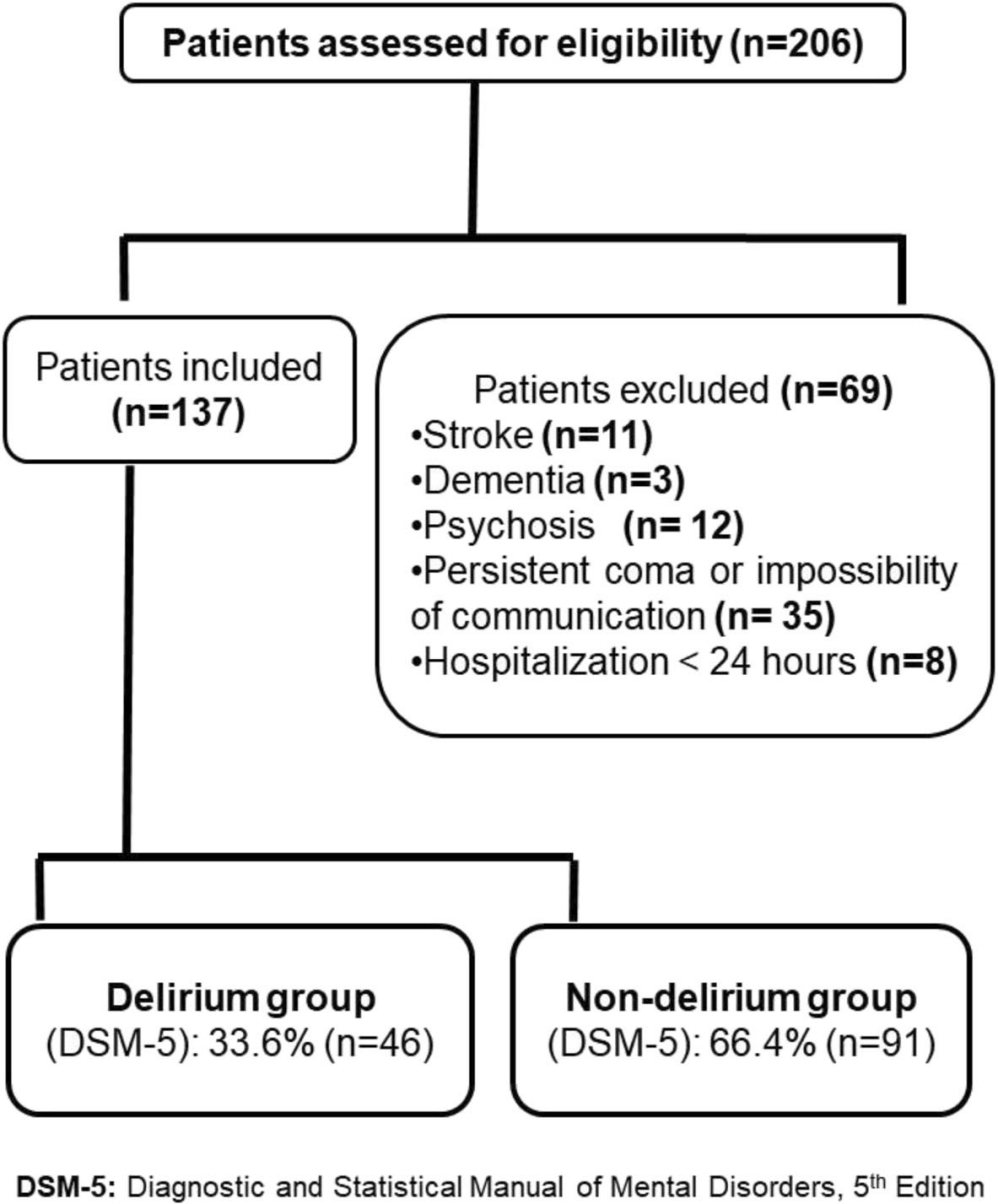
Study flow diagram

Among the 206 recruited patients, 137 were included (Fig. 1). Table 1 shows their main characteristics. Using the DSM-V criteria evaluations, 46 and 91 patients were diagnosed with (delirium group) and without (non-delirium group) delirium, respectively. The patients’ total sample profile was a male smoker aged 60 years, having a chronic obstructive pulmonary disease and a median of Charlson comorbidity index equals to 3.3, admitted to ICU for a respiratory disorder, and who had a severe disease (median SAPS-II at admission equals to 27). The two groups were matched for age, sex, Charlson comorbidity index, invasive mechanical ventilation, addictive behaviors, underlying diseases, and reasons for admission. However, compared to the delirium group, the non-delirium one had a significantly lower SAPS II score (Table 1).

**Table 1:** Patients’ demographic and clinical characteristics

Variables		Total sample (***n*** = 137)	Delirium^c^ group (***n*** = 46)	Non-delirium^c^ group (***n*** = 91)	***p***
**Age**^a^ (Year)		60 [49–68]	66 [47.5–77.0]	59 [52–68]	0.13
**Sex**^b^ (Male)		102 (74.5)	38 (82.6)	64 (70.3)	0.12
**Simplified Acute Physiology Score II**^a^		27 [22.0–33.5]	33.[26.2–38.2]	24 [20–32]	0.00
**Charlson index**^a^		3.3 [0–10]	4.0 [2–5]	3.0 [2–4]	0.49
**Invasive mechanical ventilation**^b^		49 (35.7)	27 (58.7)	22 (24.2)	0.00
**Addictive behaviors**^b^	Smoking	70 (51.1)	30 (65.2)	40 (44.0)	0.02
	Alcohol abuse	11 (8.0)	7 (15.2)	4 (4.4)	0.06
**Underlying diseases**^b^	Chronic obstructive pulmonary disease	74 (54.4)	28 (60.9)	46 (50.5)	0.25
	Hypertension	38 (27.7)	12 (26.1)	26 (28.6)	0.76
	Diabetes mellitus	47 (34.3)	11 (23.9)	36 (39.6)	0.07
	Cardiovascular disease	16 (11.7)	4 (8.7)	12 (13.2)	0.44
	Chronic renal failure	6 (4.4)	1 (2.2)	5 (5.5)	0.34
	Dysthyroidism	3 (2.2)	1 (2.2)	2 (2.2)	0.99
**Reasons for admission**^b^	Respiratory disorders	103 (75.2)	34 (73.9)	69 (75.8)	0.80
	Circulatory disorders	10 (7.3)	2 (4.3)	8 (8.8)	0.55
	Neurological disorders	8 (5.8)	3 (6.5)	5 (5.5)	0.81
	Metabolic disorders	9 (6.6)	5 (10.9)	4 (4.4)	0.28
	Toxic disorders	7 (5.1)	5 (5.5)	2 (4.3)	0.77

Data were: ^a^Median [interquartile range]; ^b^Number(%). Probability (p): comparison between the 2 groups (Wilcoxon-Mann-Whitney test for continuous data and chi-square test for categorical data)

^**c**^**Delirium as diagnosed by the diagnostic and statistical manual of mental Disorders (5th Edition) criteria**

### Validity: internal consistency, sensitivity and specificity

When applying the Tunisian version of the CAM-ICU, 38(27.7%) patients were diagnosed with delirium by the first rater and 45(32.6%) patients by the second one. The Cronbach’s α of the first and second raters’ evaluations using the Tunisian version of the CAM-ICU were 0.886 and 0.887, respectively.

Table 2 displays the validity of the Tunisian version of the CAM-ICU. The sensitivities of the two raters’ evaluations were 80.4% for the first rater and 95.7% for the second one. The specificity of the two raters’ evaluations was 98.9% for both. PPVs and NPVs were 97.4 and 90.9%, and 97.8 and 97.8%, respectively, for the first and the second raters.

**Table 2:** Validity and internal consistency of the Tunisian version of the Confusion Assessment Method for the Intensive Care Unit (*n* = 137)

	Sensitivity (95% CI)	Specificity (95% CI)	PPV (95% CI)	NPV (95% CI)	Internal consistency (Cronbach’ α)
**Rater 1**	80.4 (65.6–90.1)	98.9 (93.2–99.9)	97.4 (94.6–99.9)	90.9 (83.0–95.5)	0.886
**Rater 2**	95.7 (84.0–99.2)	98.9 (93.1–99.9)	97.8 (86.8–99.9)	97.8 (91.5–99.9)	0.887

*CI* confidence interval, *PPV* positive predictive value, *NPV* negative predictive value

### Reliability

Table 3 displays the inter-rater reliability of the Tunisian version of the CAM-ICU. The inter-rater reliability between the two raters in terms of assessing delirium was “almost perfect” (Cohen’s κ = 0.844, *p* < 0.001). The lowest kappa value was 0.648 for feature-3 (disorganized thinking).

**Table 3:** Inter-rater reliability of the Tunisian version of the Confusion Assessment Method for the Intensive Care Unit (CAM-ICU)

	Kappa	Probability
**Feature-1** Acute onset or fluctuating course of the mental status	0.839	< 10^−3^
**Feature-2** Inattention	0.818	< 10^−3^
**Feature-3** Disorganized thinking	0.648	< 10^− 3^
**Feature-4** Altered level of consciousness	0.877	< 10^− 3^
**Overall CAM-ICU**	0.844	< 10^−3^

## Discussion

The Tunisian version of the CAM-ICU showed good validity and reliability in detecting delirium in Tunisian critically ill patients. It “appears” to be sufficiently accurate as a diagnostic tool with reasonable discriminative properties. In the Tunisian ICUs, delirium diagnoses rely only on clinical impressions. To the best of the authors’ knowledge, this is the first study that validated a Tunisian/Maghrebi version of the CAM-ICU. In the Arab world, only two similar studies were performed in Egypt and Saudi Arabia [14, 15].

The back-translation and monolingual test method was applied in this study as it is the most common and highly recommended procedure for translating [24]. This technique helps to ensure equivalence between the original and the target language versions. The sample size calculated according to a predictive eq. [17], seems to be satisfactory, compared to the sample sizes of other related studies (e.g.; the number of included patients varied from 19 [25] to 306 [26]). Similar to other reports [27–31], patients admitted for an acute stroke and those with histories of dementia or psychosis were not included. In fact, differentiating delirium from other psychiatric diseases is difficult and could lead to biased results [32]. The sample size and the heterogeneity of the population involved in this study allow the data to be generalized for critically ill patients in Tunisian ICUs.

The involved patients underwent paired evaluations with the CAM-ICU and a standard reference evaluation by a psychiatrist using the DSM-V criteria [22]. The delirium frequency noticed in this study (33.6%) is in line with that reported in previous studies [28, 33, 34]. However, this study was not designed to investigate delirium prevalence which can be higher since only diurnal evaluation was considered.

The Tunisian version of the CAM-ICU seems to have good psychometric properties. Similar results were reported in other related studies (Table 4). The Tunisian version of the CAM-ICU showed a high accuracy with sensitivities at 80.4 and 95.7%, and specificities at 98.9% (Table 2). The current findings are in line with those obtained in the CAM-ICU original version. In the original cohort study for the CAM-ICU validation, the two nurses’ and intensivist’s sensitivities, when using the CAM-ICU compared with the reference standard, were 95, 96 and 100%, respectively. Their specificities were 93, 93 and 89%, respectively [11]. Compared to other translated versions of the CAM-ICU [10, 15, 25, 34], the Tunisian version had slightly higher sensitivity and specificity than the Portuguese, the Korean, the Greek and the Egyptian versions (Table 4). The discrepancies between the studies may be partly explained by the different CAM-ICU implementation procedures, and by the heterogeneity in the methodological issues.

**Table 4:** Validation of CAM-ICU versions in the literature

1st author (Language) (Ref)	Gold standard	Sensitivity (95% CI)	Specificity (95% CI)	PPV (95% CI)	NPV (95% CI)
**Ely (English)** [11]	DSM-IV	95.0 (77.0–100.0) 96.0 (78.0–100.0) 100.0 (80.0–100.0)	93.0 (68.0–100.0) 93.0 (68.0–100.0) 89.0 (51.0–100.0)	ND	ND
**Adamis (Greek)** [10]	DSM-IV-TR	87.5 (69–95) 79.2 (59–90)	91.5 (80.0–96.0) 87.2 (75–87)	84.0 (65.0–94.0) 76.0 (56.0–88.0)	94.0 (82.0–97.0) 89.0 (77.0–89.0)
**Héo (Korean)** [30]	DSM-IV-TR	89.8 77.4	72.4 75.8	ND	ND
**Gusmao-Flores (Portuguese)** [34]	DSM-IV-TR	72.5 (55.9–84.9)	96.2 (88.5–99.0)	90.6 (73.8–97.5)	87.4 (78.1–93.2)
**Wang (Chinese)** [35]	DSM-IV-TR	91.8 (84.8–99.2) 93.4 (85.4–100)	90.8 (84.2–97.4) 87.7 (81.5–93.9)	90.3 (89.4–97.2) 87.7 (81.5–93.9)	92.2 (85.2–99.2) 93.4 (85.4–100)
**Mitasova (Czech)** [36]	DSM-IV	76.0 (54.9–90.6)	98.1 (93.2–99.8)	90.5 (69.6–98.8)	94.4 (88.3–97.9)
**Koga (Japanese)** [29]	DSM-IV-TR	83.0 (59.0–96.0) 78.0 (52.0–94.0)	95.0 (87.0–99.0) 97.0 (89.0–100.0)	83.0 (59.0–96.0) 88.0 (62.0–98.0)	97.0 (89.0–100.0) 94.0 (85.0–98.0)
**Pipanmekaporn (Thai)** [37]	DSM-IV-TR	92.3 (64.0–99.8)	94.7 (85.4–98.9)	80.0 (51.9–95.7)	98.2 (90.3–100.0)
**van Eijk-1 (Dutch)** [38]	DSM-IV	64.0 (49.0–77.0)	88.0 (79.0–93.0)	73.0 (57.0–85.0)	83.0 (74.0–89.0)
**van Eijk-2 (Dutch)** [26]	DSM-IV-TR	47.0 (35–58)	98.0 (93–100)	95.0 (80.0–99.0)	72.0 (64.0–79.0)
**Toro (Spanish)** [39]	DSM-IV-TR	79.4 (63.2–89.7)	97.9 (92.6–99.4)	93.1 (78.0–98.1)	93.0 (86.3–96.6)
**Guenther (German)** [40]	DSM-IV	88.0 (69.0–98.0) 92.0 (74.0–99.0)	100.0 (88.0–100.0) 100.0 (88.0–100)	100.0 (85.0–100.0) 100.0 (85.0–100.0)	91.0 (75.0–98.0) 94.0 (79.0–99.0)
**Hestermann (German)** [41]	DSM-IV	77.0 (74.0–100.0) 77.0 (74.0–100.0)	96.0 (74.0–100.0) 100.0 (74.0–100.0)	91.0100.0	89.0 90.0
**Aljuaid (Saudian Arabia)** [14]	DSM-V	74.0 (63.0–84.0) 56.0 (44.0–68.0)	98.0 (93.0–100.0) 92.0 (84.0–100.0)	98.0 (94.0–100.0) 93.0 (85.0–100.0)	68.0 (56.0–81.0) 54.0 (42.0–66.0)
**Selim (Egyptian)** [15]	DSM-IV-TR	81.0 (60.0–93.0) 85.0 (65.0–95.0)	81.0 (62.0–92.0) 81.0 (62.0–92.0)	78.0 (57.0–91.0) 79.0 (60.0–91.0)	83.0 (65.0–94.0) 86.0 (67.0–95.0)
**Present study (Tunisian)**	DSM-V	80.4 (65.6–90.1) 95.7 (84.0–99.2)	98.9 (93.2–99.9) 98.9 (93.1–99.9)	97.4 (94.6–99.9) 97.8 (86.8–99.9)	90.9 (83.0–95.5) 97.8 (91.5–99.9)

*CAM-ICU* Confusion Assessment Method for the Intensive Care Unit, *CI* confidence interval, *DSM* Diagnostic and Statistical Manual of Mental Disorders, *ND* not determined, *NPV* negative predictive value, *PPV* positive predictive value. Ref: Reference *TR* Text Revision

The Inter-rater reliability between the two raters in assessing delirium was satisfactory (Table 3). Other validated versions yielded similar results [15, 27, 30, 37]. For example, the inter-rater reliability between the two raters was 0.81, > 0.81, 0.81, and 0.82 for the Korean [30], the Swedish [27], the Thai [37], and the Egyptian [15] versions, respectively.

The features’ inter-rater reliability between the two raters was also acceptable. The lowest kappa value was 0.648 for feature-3 (disorganized thinking) (Table 3). Similar results were reported in the Greek and the Korean versions [10, 30]. This disagreement between the two raters can be explained by three reasons. The first was the fluctuating nature of delirium [13]. According to Madrid-Navarro et al. [42], delirium, is linked to disorganization of the circadian system in critically ill patients. The second reason was the time frame between the two evaluations (4 h in this study). According to Gaspardo et al. [28], the agreement strength between the raters grows when paired assessments are performed within 1 h. The last reason was the effect of sedation depth on delirium assessment and how it was handled by the different raters [31].

This study has several limitations. First, although back-translation combined with both monolingual and bilingual tests is the most complete instrument for the translation procedure [24], the above method could not be used in this study because there were not enough bilingual subjects. Secondly, as in previous validation studies [11, 14, 27, 28, 30], patients with dementia and neuropsychiatric diseases were excluded. Further studies are needed to assess the psychometric properties of CAM-ICU in those patients. Thirdly, the Tunisian version of the CAM-ICU was used once a day. Given the fluctuating course of delirium, it is recommended to use the tool more frequently [43, 44]. Finally, some evaluations were performed at different moments. However, the investigators tried to minimize the time between the raters’ evaluations and the gold standard evaluation. This was not always possible due to logistic difficulties. Indeed, the time could be up to 4 hours between the CAM-ICU evaluations and the evaluation performed by psychiatrist.

## Conclusions

The Tunisian version of the CAM-ICU appears to be a valid and reliable tool for delirium detection among ventilated and non-ventilated ICU patients when compared to the gold standard psychiatrist evaluation, namely the DSM-V criteria. This delirium assessment tool can be easily incorporated in the daily clinical practice following an appropriate training.

## Data Availability

All data and materials related to this study can be obtained by contacting the corresponding author. The Tunisian version of the CAM-ICU is available at the website www.icudelirium.org

## Abbreviations

CAM-ICU: Confusion Assessment Method for the Intensive Care Unit
DSM-V: Diagnostic and Statistical Manual of Mental Disorders,5th Edition
NPV: Negative predictive value
PPV: Positive predictive value
RASS: Richmond Agitation-Sedation Scale
SAPS-II: Simplified Acute Physiological Score
SCCM: Society of Critical Care Medicine
